# Supplementary material for: Implementation of differentiated service delivery strategies for patients with tuberculosis in Haiti during a severe humanitarian crisis, January–August 2021
Source: PLOS Glob Public Health. 2026 Jul 22;6(7):e0006209. doi: 10.1371/journal.pgph.0006209 (PMC13390852; doi:10.1371/journal.pgph.0006209)
Supplement: S1 Table — (DOCX) [file pgph.0006209.s001.docx]

**S1 Table. Characteristics of patients receiving DSD TB treatment at two health facilities in Port-au-Prince, Haiti - 2022**

| **Demographic or Clinical Variable** | **2+4 Group N = 76 (%)** | **2+2+2 Group N = 75 (%)** | **p-value** |
| --- | --- | --- | --- |
| **Sex** |  |  |  |
| Female | 31 (40.8) | 23 (30.7) | 0.194 |
| Male | 45 (59.2) | 52 (69.3) |  |
| **Age** |  |  |  |
| 18-29 | 30 (39.4) | 23 (30.7) | 0.465 |
| 30-44 | 25 (32.9) | 34 (45.3) |  |
| 45-59 | 17 (22.4) | 15 (20.0) |  |
| 60+ | 4 (5.3) | 3 (4.0) |  |
| **Residential Area** |  |  |  |
| Port-Au-Prince | 28 (36.8) | 28 (37.3) | 0.950 |
| Other | 48 (63.2) | 47 (62.7) |  |
| **Highest Education Level** |  |  |  |
| No formal education | 5 (6.6) | 8 (10.7) | 0.304 |
| Basic/Primary | 14 (18.4) | 19 (25.3) |  |
| Secondary/Vocational | 51 (67.1) | 39 (52.0) |  |
| University | 3 (4.0) | 2 (2.7) |  |
| No data | 3 (4.0) | 7 (9.3) |  |
| **Income*** |  |  |  |
| No Income | 30 (39.5) | 28 (37.3) | 0.449 |
| Less than $5000/year | 33 (43.4) | 27 (36.0) |  |
| Greater than $5000/year | 10 (13.2) | 13 (17.3) |  |
| No data | 3 (3.9) | 7 (9.3) |  |
| **Facility** |  |  |  |
| IMIS | 40 (52.6) | 40 (53.3) | 0.931 |
| INLR | 36 (47.4) | 43 (46.7) |  |
| **Weight** |  |  |  |
| *No. pts w/ weight data* | *69* | *62* |  |
| <=Q1 in weight | 9 (13.0) | 17 (27.4) | 0.041 |
| >Q1 in weight | 60 (87.0) | 45 (72.6) |  |
| Median weight (IQR) | 54.2kg (49.5 - 60) | 52kg (45.0 - 60.0) | 0.201 |
| **HIV Status** |  |  |  |
| Positive | 70 (92.1) | 6 (7.9) | 0.772 |
| Negative | 70 (93.3) | 5 (6.7) |  |
| Inconclusive | 0 (0.0) | 0 (0.0) |  |
| **Type of TB** |  |  |  |
| Pulmonary | 75 (98.7) | 74 (98.7) | 0.993 |
| Extrapulmonary | 1 (1.3) | 1 (1.3) |  |
| **Patient Category** |  |  |  |
| New | 68 (89.5) | 67 (89.3) | 0.978 |
| Retreatment | 8 (10.5) | 8 (10.7) |  |
| Others | 0 (0.0) | 0 (0.0) |  |
| **Diagnosis type** |  |  |  |
| Bacteriological | 71 (93.4) | 73 (97.4) | 0.253 |
| Clinical | 5 (6.6) | 2 (2.7) |  |
| **Chest X-ray Results** |  |  |  |
| Suggestive of TB | 63 (82.9) | 63 (82.6) | 0.594 |
| Not Suggestive of TB | 0 (0.0) | 1 (1.3) |  |
| No chest X-ray | 13 (17.1) | 12 (16.0) |  |

DSD: Differentiated service delivery care; SOC: Standard of care; HIV: Human immunodeficiency virus; IMIS: Institut des Maladies Infectieuses et de la Santé de la Reproduction; INLR: Institut National de Laboratoire et de Recherches; IQR: Interquartile range; ^*^Personal income - not household income; ^**^Others: Transferred in (no clinical information to determine if new or retreatment)
