# Supplementary material for: Implementation of differentiated service delivery strategies for patients with tuberculosis in Haiti during a severe humanitarian crisis, January–August 2021
Source: PLOS Glob Public Health. 2026 Jul 22;6(7):e0006209. doi: 10.1371/journal.pgph.0006209 (PMC13390852; doi:10.1371/journal.pgph.0006209)
Supplement: S2 Table — (DOCX) [file pgph.0006209.s002.docx]

**S2 Table. Pulmonary bacteriologically-confirmed sensitivity analysis of the association between DSD TB treatment and TB treatment success at two health facilities in Port-au-Prince, Haiti – 2022**

| **Cohort** | **RR (95% CL)** | **p-value** | **aRR (95% CL)** | **p-value** | **% change** |
| --- | --- | --- | --- | --- | --- |
| Full cohort | 1.20 (1.12 - 1.29) | <0.001 | 1.10 (1.02 - 1.18) | 0.015 | - |
| PTB bacteriologically confirmed only | 1.13 (1.05 - 1.21) | 0.001 | 1.10 (1.02 - 1.19) | 0.020 | 0.15 |

RR: risk ratio; aRR: Adjusted risk ratio; CL: confidence limits; PTB: Pulmonary Tuberculosis
