# Supplementary material for: Implementation of differentiated service delivery strategies for patients with tuberculosis in Haiti during a severe humanitarian crisis, January–August 2021
Source: PLOS Glob Public Health. 2026 Jul 22;6(7):e0006209. doi: 10.1371/journal.pgph.0006209 (PMC13390852; doi:10.1371/journal.pgph.0006209)
Supplement: Attachment — Submitted filename: PGPH-D-26-00962_reviewer.pdf [file pgph.0006209.s005.pdf]

PLOS Global Public Health

Implementation of differentiated service delivery strategies for patients with tuberculosis in Haiti during a severe humanitarian crisis, January – August 2021

--Manuscript Draft--

Manuscript Number:	PGPH-D-26-00962
Article Type:	Research Article
Full Title:	Implementation of differentiated service delivery strategies for patients with tuberculosis in Haiti during a severe humanitarian crisis, January – August 2021
Short Title:	Implementing DSD for TB care in Haiti
Corresponding Author:	Macarthur Charles<br>Centers for Disease Control and Prevention<br>Atlanta, UNITED STATES OF AMERICA
Order of Authors:	Stalz Charles Vilbrun
	Christina Braccio
	Gerry Marcel Augustin
	Kesner François
	Milo Richard
	Patrice Joseph
	Jennifer B Harris
	Macarthur Charles
Keywords:	
Abstract:	Global efforts to control tuberculosis (TB) faced significant challenges during the COVID-19 pandemic, particularly in Haiti, where it coincided with a severe humanitarian crisis. To minimize facility visits for patients, a differentiated service delivery (DSD) model with multi-month dispensing of TB drugs was implemented at two high-volume health facilities in Port-au-Prince, Haiti. All patients aged 18 years and older who initiated drug-susceptible TB treatment from January 1 to August 31, 2022 were included in the prospective cohort. Clinicians first identified clinically stable patients who met criteria for DSD eligibility and offered them enrollment in the DSD model. Those deemed unsuitable for DSD or declined DSD were treated under the standard of care (SOC) model. DSD patients received an initial 2-month supply of medication followed by either a 4-month supply or two additional 2-month supplies. They also received remote follow-up via mobile communication within 24–48 hours of treatment initiation, biweekly during the first two months, and monthly during the last four months. Under SOC, patients came to the facility for clinical review and drug dispensation every 2–4 weeks during the first two months, and monthly during the last four months. Robust Poisson regression was used to analyze the relationship between treatment success and cohort type.During the project period, a total of 551 patients initiated TB treatment: 151 in the DSD model and 400 under SOC. Thirty-nine percent were female; median age was 33 years; and 25% were HIV-positive. Treatment success (defined as those who were cured or completed treatment) was significantly higher in the DSD group at (140/151; 93%), compared to 312/400 (78%) in the SOC group (adjusted risk ratio [aRR]: 1.10, p=0.02): however, there was no significant difference after excluding patients who were lost-to-follow-up (aRR: 1.00; p=0.85).This project demonstrates that a DSD model can achieve high TB treatment success rates, even amid challenging circumstances like the COVID-19 pandemic and socio-political unrest. Adapting healthcare systems through innovative service delivery methods is essential to effectively meet patient needs during global health crises.
Additional Information:	
Question	Response
Financial Disclosure	This project evaluation was funded and sponsored by the US Centers for Disease

Powered by Editorial Manager® and ProduXion Manager® from Aries Systems Corporation

Enter a financial disclosure statement that describes the sources of funding for the work included in this submission and the role the funder(s) played. This includes grants and any commercial funding of the work or authors.<br><br>This statement will be typeset if the manuscript is accepted for publication.<br><br>*Please review the submission guidelines and the instructions link below for detailed requirements and guidance.*	Control and Prevention's (US CDC) COVID-19 Response International Task Force. The findings and conclusions presented in this manuscript are those of the authors and do not necessarily reflect the official positions of the funding agencies.
**Competing Interests**<br><br>On behalf of all authors, disclose any competing interests that could be perceived to bias this work.<br><br>This statement will be typeset if the manuscript is accepted for publication.<br><br>*Please review the instructions link below and PLOS Global Public Health's competing interests policy to determine what information must be disclosed at submission.*	The authors declare no competing interests.
**Data Availability**<br><br>Before publication, Authors are required to make fully available and without restriction all data underlying their findings. Please see our PLOS Data Policy page for detailed information on this policy.<br><br>A **Data Availability Statement**, detailing where the data can be accessed, is required at first submission. Insert your Data Availability Statement in the box	All relevant data are within the paper and its Supporting information files. We have included a de-identified dataset as supporting information.

below.<br><br>Please see the data reporting section of our submission guidelines for instructions on what you need to include in your Data Availability Statement.<br><br>This statement will be typeset if the manuscript is accepted for publication.<br><br>*PLOS allows rare exemptions to address legal and ethical concerns. If you have legal or ethical restrictions, please use the box below to detail these in full sentences for the Journal team to consider.*	

Manuscript
Click here to access/download;Manuscript;Haiti TB DSD Manuscript_final_cleared_plos formatted.docx

Implementation of differentiated service delivery strategies for patients with tuberculosis in Haiti during a severe humanitarian crisis, January – August 2021

Adapting DSD models for tuberculosis care in Haiti

Stalz Charles Vilbrun[1¶], Christina Braccio[2¶], Gerry Marcel Augustin[3], Kesner François[4], Milo Richard[5], Patrice Joseph[1], Jennifer B. Harris[2], Macarthur Charles[2*]

[1] Les Centres GHESKIO, Port-au-Prince, Haiti

[2] Division of Global HIV and Tuberculosis, US Centers for Disease Control and Prevention, Atlanta, GA, USA

[3] Division of Global HIV and Tuberculosis, US Centers for Disease Control and Prevention, Port-au-Prince, Haiti

[4] National HIV Program, Ministry of Health, Port-au-Prince, Haiti

[5] National Tuberculosis Program, Ministry of Health, Port-au-Prince, Haiti

* Corresponding author

E-mail: mcharles2@cdc.gov (MC)

¶These authors contributed equally to this work.

Abstract

Global efforts to control tuberculosis (TB) faced significant challenges during the COVID-19 pandemic, particularly in Haiti, where it coincided with a severe humanitarian crisis. To minimize facility visits for patients, a differentiated service delivery (DSD) model with multi-month dispensing of TB drugs was implemented at two high-volume health facilities in Port-au-Prince, Haiti. All patients aged 18 years and older who initiated drug-susceptible TB treatment from January 1 to August 31, 2022 were included in the prospective cohort. Clinicians first identified clinically stable patients who met criteria for DSD eligibility and offered them enrollment in the DSD model. Those deemed unsuitable for DSD or declined DSD were treated under the standard of care (SOC) model. DSD patients received an initial 2-month supply of medication followed by either a 4-month supply or two additional 2-month supplies. They also received remote follow-up via mobile communication within 24–48 hours of treatment initiation, biweekly during the first two months, and monthly during the last four months. Under SOC, patients came to the facility for clinical review and drug dispensation every 2–4 weeks during the first two months, and monthly during the last four months. Robust Poisson regression was used to analyze the relationship between treatment success and cohort type.

During the project period, a total of 551 patients initiated TB treatment: 151 in the DSD model and 400 under SOC. Thirty-nine percent were female; median age was 33 years; and 25% were HIV-positive. Treatment success (defined as those who were cured or completed treatment) was significantly higher in the DSD group at (140/151; 93%), compared to 312/400 (78%) in the SOC group (adjusted risk ratio [aRR]: 1.10, p=0.02): however, there was no significant difference after excluding patients who were lost-to-follow-up (aRR: 1.00; p=0.85).

This project demonstrates that a DSD model can achieve high TB treatment success rates, even amid challenging circumstances like the COVID-19 pandemic and socio-political unrest. Adapting healthcare

systems through innovative service delivery methods is essential to effectively meet patient needs during global health crises.

Introduction

Global efforts to control tuberculosis (TB) faced significant challenges during the COVID-19 pandemic [1]. Patients and healthcare providers were profoundly affected, as many health facilities had limited resources and operating hours to address TB, resulting in a significant decline in patient visits. Consequently, TB case notifications decreased in several countries [2]. In Haiti, a Caribbean nation with a population of 12 million, the pandemic coincided with a severe humanitarian crisis characterized by socio-political unrest, gang violence, and kidnapping for ransom [3, 4]. These conditions pushed an already fragile healthcare system to the brink of collapse [5, 6].

Disruptions in TB diagnostic and treatment services can lead to treatment failure, increased mortality, and higher TB transmission rates within communities [7]. Therefore, it is crucial for healthcare systems and providers to ensure uninterrupted access to quality TB care and support patients throughout their entire treatment journey to minimize these disruptions and improve overall outcomes for those with TB.

Differentiated service delivery (DSD) is a patient-centered approach that restructures how health services are provided, adjusting the frequency of visits, location of care, and cadre of providers according to patient stability and context, to enhance treatment adherence, clinical outcomes, and overall health system performance in HIV and TB programs. [8]. Within global HIV treatment programs, DSD models, including multi-month dispensing (MMD) of drugs for antiretroviral therapy (ART), have been the standard of care for many years. However, TB treatment programs have adopted DSD models more slowly, given the long precedent of directly observed therapy (DOT) for TB treatment. The COVID-19 pandemic increased interest in DSD models for TB treatment as efforts were made to minimize the number of patients attending health facilities for nonurgent care. This helped to maximize resources available for COVID-19 response and minimize the risk of SARS-CoV-2 nosocomial transmission.

To mitigate the impact of the COVID-19 pandemic and the deteriorating socio-political turmoil on patient care, the Haitian Ministry of Public Health and Population (MSPP) issued interim guidance recommending alternative models of care for the delivery of essential HIV and TB services. In 2021, Les Centres GHESKIO *(Groupe Haïtien d'Etude du Sarcome de Kaposi et des Infections Opportunistes)* began implementing a comprehensive DSD care package for patients with TB that included MMD for TB medication, community or home visits, and regular phone calls.

While there is extensive documentation on positive outcomes for people living with HIV (PLHIV) who receive HIV care under DSD models [9], limited data exist regarding the implementation of these models for patients with TB. Furthermore, even less is known about patient outcomes under DSD models during complex humanitarian crises.

This project aimed to assess the outcomes of stable patients with drug-susceptible TB who received treatment in a DSD model at two health facilities in Port-au-Prince, Haiti's capital, using routinely collected and available data. We also compared outcomes for those treated in the DSD model with those who were concurrently treated under standard of care (SOC). However, the DSD model and its evaluation were designed as a programmatic intervention, with enrollment at the clinician's discretion, rather than as a study aimed at ensuring comparable groups.

Methods

Project location and population

This project was implemented by Les Centres GHESKIO, a Haitian non-governmental organization that has been offering free care for HIV and TB since its establishment in 1982. In addition to clinical services, GHESKIO conducts training and research on HIV, TB, and non-communicable diseases. The TB DSD model was implemented at two GHESKIO health facilities in Port-au-Prince – Institut National de Laboratoire et de Recherches (INLR) and Institut des Maladies Infectieuses et de la Santé de la

Reproduction (IMIS) – from January 1, 2022 to August 31, 2022. The facilities were chosen based on their high TB burden and capacity to implement the project.

Clinicians used their discretion to offer DSD to clinically stable patients 18 years and older who lived in areas accessible to community healthcare workers (CHWs) and were initiating treatment for drug-susceptible TB. A patient with TB was considered clinically stable if the patient was ambulatory, not experiencing dyspnea at rest, and did not require supplemental oxygen. Patients offered DSD could choose to receive care under the DSD or SOC model, and those who selected DSD provided verbal consent. Patients not offered treatment under the DSD model or who declined to receive care under this model received TB treatment according to SOC practices.

TB evaluation

GHESKIO providers conduct routine TB screening for all clients presenting for care; patients who report cough for more than two weeks or raise clinical suspicion for TB for other reasons, such as weight loss, fever, or night sweats, undergo chest radiography and are asked to provide sputum specimens for GeneXpert MTB/RIF testing. Patients with a GeneXpert MTB/RIF result indicating *Mycobacterium tuberculosis* complex receive a diagnosis of bacteriologically confirmed TB. Clients with negative GeneXpert MTB/RIF test results may receive a clinical TB diagnosis based on signs and symptoms of TB as well as chest radiograph findings. Diagnosed patients are initiated on anti-TB treatment at the GHESKIO facility or referred for treatment at a facility closer to the patient's home, according to the patient's preference. GHESKIO also provides voluntary counseling and testing for HIV and same-day or rapid ART initiation for clients who are HIV-positive.

Standard of care model

Under the SOC model in Haiti, TB treatment includes provision of fixed-dose combination anti-TB medications (isoniazid, rifampicin, ethambutol, and pyrazinamide) at 2- to 4-week intervals during the first two months of treatment (intensive phase) and monthly (isoniazid and rifampicin) for the remaining

four months of treatment (continuation phase), with patients presenting to the health facility for follow-up visits each time they pick up drugs. At treatment initiation, patients receive education and counseling on TB treatment-related adverse events (AEs), with instructions to contact a designated treatment supporter or present for evaluation at onset of any sign or symptom indicative of AEs. During follow-up visits, patients are evaluated for AEs, adherence, and overall response to treatment. Sputum specimens are collected for acid-fast bacilli smear microscopy at month two, month four, and month six. As part of the DOT strategy, patients are encouraged to designate a family member or friend to accompany them over the course of treatment. These treatment supporters can be relied upon to support patients with adherence, report to healthcare providers if patients miss visits, and pick up medications on behalf of patients.

DSD model of care

GHESKIO implemented TB treatment DSD as an MMD program. DSD patients were systematically assigned (1:1) to one of two models of care: a 2+4 model or a 2+2+2 model (Fig 1). Patients initiated treatment at the healthcare facility and received a 2-month dispensation of drugs for the intensive phase of treatment. At the 2-month visit, the 2+4 group received the remaining four-months of anti-TB medications, whereas the 2+2+2 group received two months of drugs. Both groups had a 4-month visit that took place at a location of their choosing in the community for sputum collection, and where the 2+2+2 group received the last two months of drugs. All patients had a facility visit at the end of treatment.

Fig 1. Differentiated Service Delivery model of TB care patient flow at two facilities in Port-au-Prince, Haiti, 2022

Like the SOC model, counseling on AEs was provided at baseline and the 2-month visit. Providers collected sputum samples and assessed AEs, adherence, and overall response to treatment at the two-, four- and six-month follow-up visits (Fig 1). In addition to the facility and community-based visits, DSD

patients had an assigned CHW that conducted remote monitoring via phone, followed by text message or WhatsApp for patients that did not answer calls to screen for AEs and reinforce adherence since the patients had fewer in-person visits. When feasible, patients were also asked to share photographs of completed medication blister packs during remote follow-up as an additional measure of adherence verification. All patients received remote monitoring within 24-48 hours of treatment initiation, biweekly during the intensive phase, and monthly during the continuation phase. Patients also received a pre-appointment call 24–48 hours before their month two and six clinic visits.

For patients in both DSD models, planned facility visits were sometimes replaced by CHWs meeting with patients in the community, and CHWs were sent to patients' homes if patients missed visits and could not be reached by phone. However, these community and home-based visits were only feasible in neighborhoods of Port-au-Prince that were considered safe and accessible to CHWs.

Under routine program conditions, patients were expected to remain in their initial model of care (DSD or SOC) unless they transferred facilities, were lost to follow-up (LTFU), or required a clinical change in treatment. During the observation period, no patients were documented as crossing over between models; therefore, analyses were conducted according to the initial model assignment.

Data collection, abstraction, and analysis

TB treatment data, including non-identifiable demographic information, diagnostic indicators, treatment start and end dates, and treatment outcomes, were documented using the GHESKIO electronic medical record (EMR). Deidentified data were retrospectively extracted from the EMR between March 1, 2023 and August 31, 2023. Throughout the data extraction process, investigators did not have access to any information that could directly identify individual participants. All data were anonymized, and no identifiable information was included in the analysis or reporting of results.

For TB treatment outcomes, patients were considered "cured" if they were bacteriologically diagnosed and had a negative sputum smear by microscopy at the end of treatment. Patients with an outcome of "treatment completed" were those who completed the full course of treatment but were either clinically diagnosed or were bacteriologically diagnosed but did not provide a sputum sample at the end of treatment. Patients with an outcome of "treatment failure" were smear positive at the end of treatment. Patients were given a "lost to follow-up" outcome after no contact with the health facility for more than two months. Successful treatment outcomes were defined as cured and treatment completed; adverse treatment outcomes were defined as died, treatment failure, and lost to follow-up.

DSD follow-up and self-reported adherence data were collected from project-specific forms used for remote follow-up. Adherence data from the SOC cohort and the DSD facility visits were not available.

After extraction from the GHESKIO EMR and project databases, data were imported into SAS 9.4 for analysis. Patient demographic and clinical characteristics were summarized with descriptive analyses. Associations between treatment cohort (DSD or SOC) and relevant variables were assessed using Pearson χ^2 tests. Since no significant differences in clinical characteristics or treatment outcomes were identified between the 2+2+2 and 2+4 subgroups, analyses focused on the full DSD cohort to increase statistical power.

Robust Poisson regression with a log link was used to examine the relationship between TB treatment cohort, potential confounders, and treatment success. This approach directly estimates risk ratios and provides robust standard errors to account for potential variance in misspecification. Weight was categorized as less than or greater than the 25th percentile within each sex because height was not available to calculate body mass index. Potential confounders with a p-value < 0.2 in univariable analyses were considered for multivariable models using a forward selection approach to ensure model

stability and reduce the risk of overfitting given the relatively small sample size. Statistical significance was set at $\alpha = 0.05$ for associations between treatment group and outcomes.

Model stability and risk of overfitting were assessed by comparing the forward-selection model with alternative specifications including all prespecified covariates. Goodness-of-fit for GEE Poisson models was evaluated using the quasi-likelihood under the independence model criterion (QIC) and its corrected form (QICu). Lower QIC values were used to identify the best-fitting and most parsimonious model. Model adequacy was further supported by low collinearity (all variance inflation factors <2) and stability of adjusted risk ratios across alternative model specifications. The final multivariable model was selected based on consistency of effect estimates, lower QIC values, and acceptable goodness-of-fit.

Given the limited size in the DSD cohort, we assessed the precision of key contrasts using observed event rates. Power calculations indicated that the comparison of composite treatment success (92.7% vs 78.0%) had >99% power to detect the observed 14.7 percentage-point (pp) difference, whereas the analysis excluding patients lost to follow-up (95.3% vs 93.7%) had approximately 11% power to detect the observed 1.6 pp difference, indicating limited ability to identify small differences in this subgroup. The comparison of bacteriologic cure alone (58.3% vs 37.0%) had >99% power to detect the observed 21.3 pp difference. To communicate precision, absolute risk differences with 95% confidence intervals were also calculated.

Sensitivity analyses were conducted to explore the robustness of the primary findings. First, patients who were LTFU were excluded because LTFU was rare in the DSD cohort and may reflect selection bias or enhanced follow-up support. A second sensitivity analysis restricted the cohort to bacteriologically confirmed pulmonary TB (PTB); because effect estimates were similar to the full cohort, no further PTB-only analyses were pursued.

To evaluate whether differences in HIV status affected the observed association between DSD and treatment success, an additional HIV-stratified sensitivity analysis was conducted. Separate robust Poisson regression models were fit among HIV-negative and HIV-positive patients. Because HIV status did not vary within each stratum, HIV was omitted as a covariate in the stratified models while all other covariates from the primary multivariable model were retained. These analyses evaluated both the robustness of the primary findings and potential effect modification by HIV status.

Ethical considerations

The protocol was reviewed and approved by the Haiti National Bioethics Committee (Reference number: 2223-7). This activity was reviewed by CDC, deemed not research, and was conducted consistent with applicable federal law (45 C.F.R. part 46, 21 C.F.R. part 56; 42 U.S.C. §241(d); 5 U.S.C. §552a; 44 U.S.C. §3501 et seq.) and US CDC policy. The requirement for written informed consent was waived. Instead, participants provided verbal informed consent after receiving a thorough explanation of the TB DSD model of care, including its associated risks and benefits. This verbal consent process was documented on a verbal consent form and witnessed by the study coordinator.

Results

Demographic and clinical characteristics of cohorts

A total of 551 patients initiated TB treatment between 1 January and 31 August, 2022. The DSD cohort included 151 patients, while the SOC cohort included 400 patients. In the DSD cohort, 54 (35.8%) patients were female and median age was 34 years (interquartile range [IQR]: 27-45). Seventy-six (50.3%) were enrolled in the 2+4 model, and 75 (49.7%) in the 2+2+2 model. In the SOC cohort, 161 (40.2%) patients were female and median age was 33 years (IQR: 24-43).

Fewer of the DSD cohort (37.1%) resided in Port-au-Prince than in the SOC cohort (51.7%; p=0.02) and personal income distributions differed (p=0.03) (Table 1). A greater proportion of the DSD cohort (95.4%) was bacteriologically diagnosed than the SOC cohort (68.5%; p<0.0001); fewer DSD patients

(7.2%) than SOC patients (31.8%) were HIV positive (p<0.0001); and more of the DSD cohort (98.7%) had pulmonary TB than the SOC cohort (92.0%; p=0.004). Among patients with a documented weight (n=496), median weight was higher in the DSD group (53.5 kg, IQR: 46.0-60.0 kg) than in the SOC group (50.0 kg, IQR: 44.0-57.0 kg; p=0.03). Additional characteristics are shown in Table 1.

Table 1. Characteristics of patients receiving TB treatment at two health facilities in Port-au-Prince, Haiti - 2022

Characteristic	All patients (n = 551) n (%)	DSD cohort (n = 151) n (%)	SOC cohort (n = 400) n (%)	p-value
**Sex**				
Female	215 (39.0)	54 (35.8)	161 (40.2)	0.335
Male	336 (61.0)	97 (64.2)	239 (59.8)	
**Age**				
18-29	215 (39.0)	53 (35.1)	162 (40.5)	0.672
30-44	203 (36.8)	59 (39.3)	144 (36.0)	
45-59	106 (19.2)	32 (21.3)	74 (18.5)	
60+	27 (4.9)	7 (4.7)	20 (5.0)	
**Residential Area**				
Port-au-Prince	302 (54.8)	56 (37.1)	207 (51.7)	0.019
Other	249 (45.2)	95 (62.9)	193 (48.3)	
**Highest Education Level**				
No formal education	134 (24.3)	13 (8.6)	46 (11.5)	0.250
Basic/Primary	59 (10.7)	33 (21.9)	101 (25.3)	
Secondary/Vocational	287 (52.1)	90 (59.6)	197 (49.4)	
University	19 (3.5)	5 (3.3)	14 (3.5)	
No data	54 (9.4)	10 (6.6)	42 (10.5)	
**Income***				
No Income	256 (46.5)	58 (38.4)	198 (49.5)	0.030
Less than $5000/year	176 (31.9)	60 (39.7)	116 (29.0)	
Greater than $5000/year	70 (12.7)	23 (15.2)	47 (11.6)	
No data	49 (8.9)	10 (6.6)	39 (9.6)	
**Facility**				
IMIS	165 (30.0)	80 (53.0)	85 (21.3)	<0.001
INLR	386 (70.0)	71 (47.0)	315 (78.8)	
**Weight**				
Number with available weight	*n = 496*	*n = 131*	*n = 365*	
<=Q1 in weight	129 (26.0)	26 (19.9)	103 (28.2)	0.061

>Q1 in weight	367 (74.0)	105 (80.1)	262 (71.8)	
Median weight kg (IQR)	51.0 (45.0 - 58.0)	53.5 (46.0 - 60.0)	50.0 (44.0 - 57.0)	0.003
**HIV Status**				
Positive	138 (25.0)	11 (7.2)	127 (31.8)	<0.001
Negative	406 (73.7)	140 (92.7)	266 (66.5)	
Inconclusive	7 (1.3)	0 (0)	7 (1.8)	
**Type of TB**				
Pulmonary	517 (93.8)	149 (98.7)	368 (92.0)	0.004
Extrapulmonary	34 (6.2)	2 (1.3)	32 (8.0)	
**Patient Category**				
New	470 (85.3)	135 (89.4)	335 (83.8)	0.131
Retreatment	75 (13.6)	16 (10.6)	59 (14.8)	
Others[**]	6 (1.1)	0 (0)	6 (1.5)	
**Diagnosis Type**				
Bacteriological	418 (75.9)	144 (95.4)	274 (68.5)	<0.001
Clinical	133 (24.1)	7 (4.6)	126 (31.5)	
**Chest X-ray Results**				
Suggestive of TB	462 (83.8)	125 (82.8)	337 (84.3)	0.724
Not Suggestive of TB	5 (0.9)	1 (0.7)	4 (1.0)	
No chest X-ray	84 (15.2)	25 (16.5)	59 (14.7)	

DSD: Differentiated service delivery care; SOC: Standard of care; HIV: Human immunodeficiency virus; IMIS: Institut des Maladies Infectieuses et de la Santé de la Reproduction; INLR: Institut National de Laboratoire et de Recherches; IQR: Interquartile range; [*]Personal income - not household income; [**]Others: Transferred in (no clinical information to determine if new or retreatment).

DSD cohort follow-up monitoring and self-reported adherence

Remote follow-up via phone was most successful on days 1 (90.0% of patients reached) and 2 (94.0%), and lowest at month 3 (78.9%) (Table 2). Across all other remote follow-up periods, the proportion of patients reached ranged from 82.6% - 86.8%. Self-reported adherence was lowest at week 2 (6.3% of patients reported missing a dose since last monitoring call). At all other time periods, the proportion of patients who reported missing a dose since the last timepoint ranged from 0.0% to 2.8%.

Table 2. Remote follow-up and self-reported adherence during TB Treatment received in a differentiated service delivery model at two facilities in Port-au-Prince, Haiti – 2022

Monitoring Phase	Total number of patients in DSD cohort*	Number reached for follow-up n (%)	Number reporting ≥1 missed dose since last timepoint** n (%)
Day 1	151	136 (90.1)	0 (0.0)
Day 2	151	142 (94.0)	1 (1.0)
Week 2	151	131 (86.8)	7 (6.3)
Week 4	150	129 (86.0)	2 (1.9)
Week 6	150	127 (84.7)	3 (2.7)
Month 3	147	116 (78.9)	0 (0.0)
Month 4	144	121 (84.0)	3 (2.8)
Month 5	144	119 (82.6)	0 (0.0)

DSD: Differentiated service delivery care; *the number of patients in care decreased over time as patients died, were lost to follow-up, or stopped treatment; **out of the number of patients who were reached at that timepoint

Treatment Outcomes

In the DSD cohort, 140 (92.7%) patients had successful treatment outcomes; 88 (58.3%) were cured and 52 (34.4%) completed treatment (Table 3). Ten (6.6%) patients had an adverse treatment outcome: 5 (3.3%) died, 1 (0.7%) had treatment failure, and 4 (2.6%) were lost to follow-up (LTFU). In the SOC cohort, 312 (78.0%) patients had a successful treatment outcome: 148 (37.0%) were cured and 164 (41.0%) completed treatment. Eighty-six (21.5%) had an adverse treatment outcome: 19 (4.8%) died and 67 (16.8%) were LTFU.

Table 3. TB treatment outcomes among patients treated under differentiated service delivery and standard of care models at two health facilities in Port-au-Price, Haiti – 2022

	All Patients		Excluding Patients who were LTFU	
Treatment Outcomes	DSD cohort N = 151 (%)	SOC cohort N = 400 (%)	DSD cohort N = 147 (%)	SOC cohort N = 333 (%)
Treatment success	140 (92.7)	312 (78.0)	140 (95.3)	312 (93.7)
Cured	88 (58.3)	148 (37.0)	88 (59.8)	148 (44.4)
Treatment Completed	52 (34.4)	164 (41.0)	52 (35.4)	164 (49.3)
Adverse outcome	10 (6.6)	86 (21.5)	6 (4.1)	19 (5.7)
Died	5 (3.3)	19 (4.8)	5 (3.4)	19 (5.7)
Failed	1 (0.7)	0 (0.0)	1 (0.7)	0 (0.0)
Lost to follow-up	4 (2.6)	67 (16.8)	-	-
Excluded from outcome analysis	1 (0.7)	2 (0.5)	1 (0.7)	2 (0.6)
Transferred out	0 (0.0)	2 (0.5)	0 (0.0)	2 (0.6)

MDR-TB diagnosis	1 (0.7)	0 (0.0)	1 (0.7)	0 (0.0)

LTFU: Lost to follow-up; DSD: Differentiated service delivery care; SOC: Standard of care; MDR-TB: Multidrug resistant tuberculosis

When LTFU outcomes were excluded, the proportion of patients with each outcome shifted. One-hundred-forty (95.3%) DSD patients had successful outcomes, while 6 (4.1%) had adverse outcomes (5 [3.4%] died and 1 [0.7%] had treatment failure). In the SOC cohort, 312 (93.7%) had a successful treatment outcome and 19 (5.7%) died.

Comparing treatment success among all patients, patients in the DSD cohort were 10% more likely to have treatment success compared to patients in the SOC cohort (adjusted Risk Ratio (aRR)=1.10; 95% CL: 1.02-1.19; p = 0.02, E-value = 1.43), after adjusting for age, sex, facility, weight, HIV status, patient category, and diagnosis type. After excluding patients who were LTFU, the adjusted association between DSD and SOC was no longer statistically significant (aRR = 1.00; 95% CL: 0.96–1.05; p = 0.85; E-value = 1.00) (Table 4). However, the absolute difference in treatment success between DSD and SOC narrowed to 1.6% (95.3% vs 93.7%), a difference for which the study had approximately 11% power to detect

Table 4. Relative risk of treatment success for patients receiving TB treatment under a differentiated service delivery model compared to those treated under the standard of care at two facilities in Port-au-Prince, Haiti – 2022.

All Patients				
Variable	RR (95% CL)	p-value	aRR[*] (95% CL)	p-value
DSD[†] vs. SOC[††]	1.20 (1.12 - 1.29)	<0.001	1.10 (1.02 - 1.19)	0.015

Excluding Patients who were LTFU[‡]				
Variable	RR (95% CL)	p-value	aRR[**] (95% CL)	p-value
DSD vs. SOC	1.02 (0.98 - 1.07)	0.381	1.00 (0.96 - 1.05)	0.849

RR: Risk ratio; aRR: Adjusted risk ratio; CL: Confidence limits; DSD: Differentiated service delivery care; SOC: Standard of care; LTFU: Lost to follow-up; [*]Adjusted for age, sex, facility, HIV status, weight, patient category, and diagnosis type; [**]Adjusted for HIV status and patient category. Thirteen patients excluded (7 with indeterminate/unknown HIV status and 6 who transferred in from another health facility and history of TB diagnosis could not be determined).

In HIV-stratified sensitivity analyses, the adjusted association between DSD participation and treatment success remained identical to the primary estimate among HIV-negative patients (aRR = 1.10; 95% CI: 1.02–1.18; 0% change). Among HIV-positive patients, the adjusted association was attenuated and imprecise (aRR = 1.04; 95% CI: 0.73–1.48; −5.5% change), reflecting small sample size. These results suggest that the observed benefit of DSD is not driven solely by differences in HIV status. (Table 5).

Table 5. HIV-stratified sensitivity analysis of the association between DSD TB treatment and TB treatment success at two health facilities in Port-au-Prince, Haiti – 2022

Cohort	RR (95% CL)	p-value	aRR (95% CL)	p-value	% change
Full cohort	1.20 (1.12 - 1.29)	<0.001	1.10 (1.02 - 1.18)	0.015	-
HIV-negative only	1.13 (1.06 - 1.22)	0.001	1.10 (1.02 - 1.18)	0.016	0.00%
HIV-positive only	1.33 (1.07 - 1.67)	0.010	1.04 (0.73 - 1.48)	0.821	5.45%

RR: Risk ratio; aRR: Adjusted risk ratio; CL: confidence limits; DSD: Differentiated service delivery care; HIV: Human immunodeficiency virus

Discussion

This project demonstrated that patients receiving TB treatment in a DSD model with MMD can achieve high treatment success rates, even in the context of the COVID-19 pandemic and socio-political unrest. Over 92% of patients receiving DSD care had successful treatment outcomes, which was higher than the national treatment success rate of 85% among patients initiating treatment for drug-sensitive TB in Haiti in 2022 [10]. Among patients evaluated in this project, treatment success was significantly higher (aRR=1.10, p=0.02) among patients treated under DSD than among patients treated under SOC.

LTFU was notably lower in the DSD (2.0%) than in the SOC cohort (15.5%). This may be partially due to improved retention through decreased barriers to drug refills, as has been observed with DSD models for antiretroviral therapy (ART) for HIV care [11]. However, because assignment to DSD versus SOC was not randomized, this difference may also be attributable to enhanced follow-up of DSD patients through a project with additional funding and CHW support that was restricted to neighborhoods considered safe and accessible for CHWs. Health care providers may also have intentionally enrolled patients anticipated to have good treatment compliance for the DSD model, recognizing those patients could safely be managed with less frequent in-person follow-up. In addition, patients who elected to participate in DSD may have differed systematically from those who remained in SOC, including in motivation or ability to engage in care. While these factors limit causal inference regarding the effect of DSD on LTFU, they also reflect a strength of the DSD approach, in which HCWs can triage patients based on clinical stability and context, channeling patients to care models that balance their needs and system capacity. DSD models enable programs to match patients to the model best suited to their clinical and contextual needs. This targeted and adaptive approach reflects the essence of differentiated care, focusing resources where they are most needed while preserving quality for all.

This project also demonstrated that remote patient monitoring through a combination of phone calls and text or WhatsApp messaging was feasible, with over 90% of patients reached during the first two days after treatment initiation and 79-87% reached at later time points. Project staff noted that remote monitoring can require additional staff or staff time depending on caseload, and that younger patients frequently provided multiple contact options, such as social applications like WhatsApp.

Self-reported adherence was very high in the DSD cohort, with 93-100% of respondents at each time point reporting no missed doses since their prior interaction with a health worker. Although self-reported adherence has inherent limitations, the innovation implemented by project staff of requesting pictures of their completed blister packs, when feasible, may have strengthened adherence verification.

These findings align with prior evidence demonstrating the successes of person-centered approaches for ART and for TB preventive treatment (TPT). Patients enrolled in DSD models for ART have higher rates of retention and viral suppression than those not in DSD models, and have a significant reduction in missed appointments and lapses in treatment [11]. Similarly, patients enrolled in TB Preventive Treatment (TPT) DSD models have reported high levels of satisfaction with the programs, and greater than 90% completion rates [12, 13]. To our knowledge, there is only limited evidence on the application of these models for TB treatment [14-18], underscoring the need for data to inform the adaptation and scale-up of DSD strategies for TB treatment, particularly in settings with high TB and HIV burden, where integrated and efficient care delivery is essential.

Transitioning to MMD models for TB treatment may be a more challenging paradigm shift than it was for ART, given the historical emphasis on DOT for TB. DOT has long been a cornerstone of TB management, particularly in low-resource settings, as it can ensure adherence to treatment regimens and has been effective in treating drug-sensitive TB [19]. But DOT often demands significant human resources, and in settings with high TB burdens and low resources, this can place a considerable strain on healthcare

workers. For patients, DOT can create multiple barriers that affect their willingness to comply, such as frequent and costly transport and visits to healthcare facilities [20].

To help patients access TB treatment, global TB guidance has prioritized patient-centered care and DOT has moved from facility-based to community and remote models [21, 22]. In the context of COVID-19, HIV programs pushed to decrease patient contact with health facilities to reduce the risk of COVID-19 transmission for both patients and providers, and many countries adapted their MMD policies for ART to expand use or intensified scale-up [23]. The results, as well as those of other pilot projects of DSD for TB treatment [14, 15, 24], suggest that MMD models for TB treatment could effectively mitigate disruptions to patient care.

Limitations

Despite the promising results observed in the DSD cohort, several limitations merit consideration. The project's programmatic nature meant that clinicians exercised discretion in offering participation in the DSD model to patients, introducing the potential for selection bias, as individuals perceived to be more stable or adherent, or those more able to engage in care, may have been preferentially enrolled in the DSD cohort, causing demographic and clinical differences between the cohorts. While analyses adjusted for measured confounders, residual and unmeasured confounding may have influenced the observed associations, there is likely still unmeasured confounding.

Because the DSD cohort was likely "healthier", it is unknown if less stable patients would have similar outcomes in a DSD model. Investigating how different patient populations respond to DSD interventions will be important for refining strategies and ensuring they meet the needs of different patients and settings.

Adherence data were not measured in the SOC cohort, so the impact of adherence on outcomes could not be assessed. In addition, the DSD model had additional project resources that were not available

under the SOC model, including CHWs and transportation for patient follow-up. This may have improved outcomes in the DSD cohort beyond what would have been achieved without additional resources, especially in a context of challenging patient follow-up due to the socio-political instability in Haiti [25, 26].

Furthermore, HIV-related clinical information was limited, restricting our ability to assess how HIV co-infection, ART status, or immunologic markers may have influenced treatment outcomes. Although HIV-stratified sensitivity analyses indicated that the DSD effect estimate was stable among HIV-negative patients, the small number of HIV-positive patients resulted in imprecise effect estimates; therefore, the non-significant association observed in this restricted cohort reflects limited precision rather than evidence of equivalence between models of care. Given the high burden of TB/HIV co-infection in similar settings, more detailed HIV data are essential for understanding whether DSD models perform consistently across subgroups with differing HIV statuses and stages.

Further evaluation is warranted to explore treatment outcomes associated with DSD models beyond a project context, particularly during complex humanitarian crises like those in Haiti. Qualitative assessments of both patient and provider perspectives would facilitate a better understanding of the acceptability and feasibility of scaling up DSD models for TB treatment.

Conclusion

Our analysis underscores the potential benefits and feasibility of implementing DSD models for TB treatment amidst challenging circumstances such as the COVID-19 pandemic and ongoing socio-political unrest in Haiti. In addition, the findings suggest that health care workers can successfully triage patients into care models requiring less frequent in-person clinic follow-up. DSD models need not apply universally; rather, their value lies in enabling programs to match patients with the care model that best fits their stability, circumstances, and preferences. By offering a more flexible and patient-centered

approach to care, these models can help ensure uninterrupted access to quality TB services. As we continue to navigate global health challenges, healthcare systems may need to adapt and innovate their service delivery methods to meet the needs of all patients effectively.

Acknowledgements

We extend our heartfelt gratitude to the dedicated nurses at the IMIS TB clinic and field workers for their invaluable commitment and resilience in successfully implementing this project amid the significant challenges in Port-au-Prince.

Author Contributions

Conceptualization: MR, PJ, GMA, JBH, MC

Data Curation: SCV, CB, JBH, MC

Formal Analysis: CB, JBH, MC

Funding Acquisition: MR, PJ, KF, JBH, MC

Investigation: SCV, CB

Methodology: SCV, CB, JBH, MC

Project Administration: CB, SVC, PJ, JBH, MC

Resources: PJ, JBH, MC

Software: CB

Supervision: KF, MR, PJ, JBH, MC

Validation: CB, JBH, MC

Visualization: CB, JBH, MC

Writing – Original Draft Preparation: CB, JBH, MC

Writing – Reviewing & Editing: SCV, CB, GMA, KF, MR, PJ, JBH, MC

SVC – Stalz Charles Vilbrun

CB – Christina Braccio

GMA – Gerry Marcel Augustin

KF – Kesner Francois

MR – Milo Richard

PJ – Patrice Joseph

JBH – Jennifer B. Harris

MC – Macarthur Charles

References

1. Marco MH, Ahmedov S, Castro KG. The global impact of COVID-19 on tuberculosis: A thematic scoping review, 2020-2023. PLOS Glob Public Health. 2024;4(7):e0003043.
2. Gunsaru V, Henrion MYR, McQuaid CF. The impact of the COVID-19 pandemic on tuberculosis treatment outcomes in 49 high burden countries. BMC Med. 2024;22(1):312.
3. PAHO. PAHO Situation Report N.6. Haiti Humanitarian Situation. . 2024 May 6, 2024.
4. World Report 2022, Events of 2021. USA: Human Rights Watch; 2022.
5. Medeus AJR, Pierre CJ, Merilan LLS, Vertilus S. Impact of gang violence in Haiti on healthcare delivery and medical education. Lancet Reg Health Am. 2024;36:100811.
6. Hauteville J-M. Haiti's health system, the victim of gang ransakcing, threatens to collapse. Le Monde. 2024 August 14, 2024;Sect. World.
7. Alipanah N, Jarlsberg L, Miller C, Linh NN, Falzon D, Jaramillo E, et al. Adherence interventions and outcomes of tuberculosis treatment: A systematic review and meta-analysis of trials and observational studies. PLoS Med. 2018;15(7):e1002595.
8. Grimsrud A, Bygrave H, Doherty M, Ehrenkranz P, Ellman T, Ferris R, et al. Reimagining HIV service delivery: the role of differentiated care from prevention to suppression. J Int AIDS Soc. 2016;19(1):21484.
9. Long L, Kuchukhidze S, Pascoe S, Nichols B, Cele R, Govathson C, et al. Differentiated models of service delivery for antiretroviral treatment of HIV in sub-Saharan Africa: a rapid review protocol. Syst Rev. 2019;8(1):314.
10. WHO. WHO Global TB Report, Tuberculosis profile: Haiti 2025 [Available from: https://worldhealthorg.shinyapps.io/tb_profiles/?_inputs_&tab=%22tables%22&lan=%22EN%22&iso2=%22HT%22&entity_type=%22country%22.
11. IAS. Differentiated service delivery for HIV treatment: Summary of published evidence. International AIDS Society; Nov 2020.
12. Mugenyi L, Namuwenge PM, Ouma S, Bakashaba B, Nanfuka M, Zech J, et al. Isoniazid preventive therapy completion between July-September 2019: A comparison across HIV differentiated service delivery models in Uganda. PLoS One. 2024;19(1):e0296239.
13. Mapingure MP, Zech JM, Hirsch-Moverman Y, Msukwa M, Howard AA, Makoni T, et al. Integrating 3HP-based tuberculosis preventive treatment into Zimbabwe's Fast Track HIV treatment model: experiences from a pilot study. J Int AIDS Soc. 2023;26(6):e26105.
14. Melaku Z, Feleke B, T. Weyeyso, T. Letta, T. Gelibo, M. Kemal, et al. Differentiated service delivery model in the treatment of people with drug-susceptible TB: Results from a prospective cohort study in Ethiopia. The Union World Conference on Lung Health; November 2024; Bali, Indonesia: Int J Tuberc Lung Dis; 2024. p. S306.
15. Weyenga H, A. Katana, W. Tomno, E. Ngugi, M. Barasa, A. Ajiboye, et al. Outcomes of differentiated TB preventative therapy service delivery models in Nairobi slums, Kenya. The Union Conference on Lung Health; Paris, France2023.
16. Okon U, Jwanle P, Nwagagbo F, Edigha E, Okoye M, Braccio C, et al. Implementation of differentiated service deliver models for TB treatmetn in North Central Nigeria. The Union World Conference on Lung Health; Paris, France: Int J Tuberc Lung Dis; 2023. p. S577.
17. Ferroussier-Davis O, Lukoye D, Alwedo S, Mudiope MN, Nalunjogi J, Kirenga JB, et al. Introducing differentiated service delivery models for tuberculosis treatment: a pilot project to inform national policy in Uganda. J Int AIDS Soc. 2025;28 Suppl 3(Suppl 3):e26483.
18. Mulenga HB, Moore BK, Lyembele C, Kalunkumya E, Sinkala T, Mwiinga L, et al. Differentiated service delivery for TB treatment. Int J Tuberc Lung Dis. 2026;30(3):107-13.

19. WHO. WHO consolidated guidelines on tuberculosis: module 4: treatment: drug-susceptible tuberculosis treatment. WHO; 2022.
20. Metcalfe JZ, O'Donnell MR, Bangsberg DR. Moving Beyond Directly Observed Therapy for Tuberculosis. PLoS Med. 2015;12(9):e1001877.
21. WHO. A Patient-Centred Approach to TB Care. 2018.
22. WHO. WHO Treatment of Drug-Susceptible Tuberculosis: Rapid Communication. 2021.
23. Bailey LE, Siberry GK, Agaba P, Douglas M, Clinkscales JR, Godfrey C. The impact of COVID-19 on multi-month dispensing (MMD) policies for antiretroviral therapy (ART) and MMD uptake in 21 PEPFAR-supported countries: a multi-country analysis. J Int AIDS Soc. 2021;24 Suppl 6(Suppl 6):e25794.
24. Ferroussier-Davis O, Lukoye D, Alwedo S, Nabukenya-Mudiope M, Nalunjogi J, Kabanda J, et al. Implementing differentiated service delivery models for TB care to inform national policy in Uganda The Union World Conference on Lung Health; November 2024; Bali, Indonesia: Int J Tuberc Lung Dis; 2024. p. S220.
25. Vilbrun SC, Souroutzidis A, Walsh KF, Ellis J, Guiteau C, Delva S, et al. Successful outcomes for patients with drug-resistant tuberculosis despite civil unrest and COVID-19 in Haiti. PLOS Glob Public Health. 2023;3(9):e0002356.
26. Joseph P, Sun R, Guiteau C, Jean Juste MA, Dorvil N, Vilbrun S, et al. Continuity of care during severe civil unrest with a model of community-based HIV care: a retrospective cohort study from Haiti. Lancet Reg Health Am. 2024;37:100847.

Figure

Click here to access/download;Figure;Fig 1_DSD model patient flow.docx

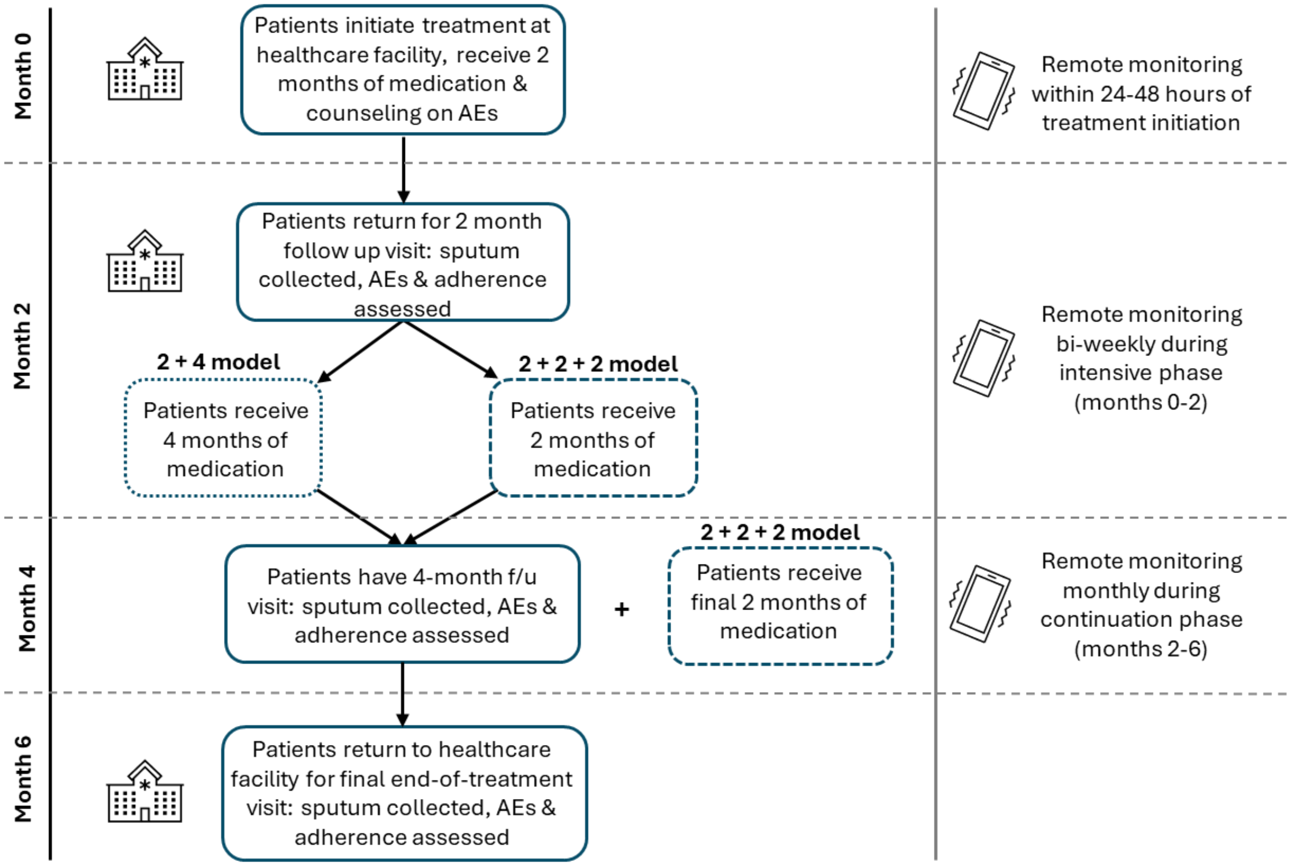

*AE: adverse event

Click here to access/download
Supporting Information
TB DSD_Supplemental Tables.docx

Click here to access/download
Supporting Information
ht_tb_dsd_final_dataset_29sep25.xlsx
