# Supplementary material for: Implementation of differentiated service delivery strategies for patients with tuberculosis in Haiti during a severe humanitarian crisis, January–August 2021
Source: PLOS Glob Public Health. 2026 Jul 22;6(7):e0006209. doi: 10.1371/journal.pgph.0006209 (PMC13390852; doi:10.1371/journal.pgph.0006209)
Supplement: Attachment — Submitted filename: Haiti_TB DSD_Response to Reviewers_clean.docx [file pgph.0006209.s007.docx]

# **Responses to Reviewers**

We thank the reviewers for their careful reading of our manuscript and for their constructive comments. We have carefully considered all of the feedback and have revised the manuscript accordingly. Their insights have helped us to clarify our methodology and strengthen our programmatic recommendations.

Below is our point-by-point response to the reviewer's general and specific comments. For ease of review, the reviewers’ original comments are provided in bold, followed by our responses in standard text. Where applicable, we have included the updated text and indicated where changes were made in the revised manuscript.

## **General Comments**

- **On the cost of scaling the DSD model:**

**Response:** Thank you for this important observation. We agree that a cost-effectiveness analysis is a crucial next step for the scalability of the DSD model. As noted in the limitations (lines 340-344), the DSD model in this project had access to additional resources, such as community health workers (CHWs) and transportation for follow-up, which were not available to the Standard of Care (SOC) model. These resources were vital to the success of the intervention, especially during a humanitarian crisis. While a detailed cost analysis was beyond the scope of this programmatic evaluation, we believe this is a critical area for future research and have incorporated it as a recommendation. Future studies should aim to conduct a formal cost-effectiveness analysis to inform national scale-up strategies.

- **On the non-randomized allocation and selection bias:**

**Response:** We appreciate the reviewer's point regarding selection bias. The manuscript acknowledges this as a key limitation (lines 92-94, 337-341). This project was designed as a programmatic intervention during a severe crisis, making a randomized controlled trial impractical. Enrollment was based on clinician discretion and patient choice, which inevitably introduced selection bias. The DSD group was clinically more stable, had fewer HIV co-infections, and higher income, among other differences. While our statistical analyses adjusted for known confounders (lines 182-189), we recognize that this cannot fully negate the bias. We agree that future research, where feasible, should aim for a more randomized allocation to better isolate the effects of the DSD model.

## **Specific Comments**

**1. Patients in the DSD group were clinically stable, less likely to be HIV positive, had more people with income, higher mean body weight and lived further from the facility. Will these differences in characteristics influence treatment outcomes?**

**Response:** This is an excellent point. We recognized that the significant baseline differences between the DSD and SOC cohorts could influence treatment outcomes. To address this, we conducted multivariable analysis using a robust Poisson regression model to adjust for potential confounders. The final analysis was adjusted for age, sex, facility, weight, HIV status, patient category, and diagnosis type (lines 182-189, 273-280, footnote of Table 4). Despite these adjustments, we found that patients in the DSD cohort were still 10% more likely to have a successful treatment outcome (aRR=1.10; 95% CI: 1.02-1.19). While this suggests that the DSD model has benefits even after accounting for the healthier patient profile at baseline, we further acknowledge in the paper that there may still be unmeasured confounding and do sensitivity analyses to further assess the relationship.

**2. DSD patients could opt in or out of participation in DSD. Do we know why patients opted out of the DSD? Please add if this information is available.**

**Response:** Thank you for pointing out this important question. We state in the manuscript that patients could decline to receive care under the DSD model (lines 97-98). However, this was a retrospective review of programmatic data, and the specific reasons for patients declining the DSD model were not systematically collected. This is a valuable point for consideration, and we recommend that future DSD implementations should include a qualitative component to better understand patient preferences and the reasons for their choices.

**3. The proportion of people with TB who were bacteriologically confirmed is phenomenally high. Can you explain why?**

**Response:** We appreciate the reviewer's attention to this detail. The high rate of bacteriologic confirmation (95.4% in the DSD cohort and 68.5% in the SOC cohort) is attributable to the specialized diagnostic capacity at the GHESKIO Centers. GHESKIO serves as a major national referral center for tuberculosis and houses the only accredited biosafety level 3 laboratory in Haiti, possessing the capacity to perform mycobacterial cultures. Furthermore, as described in the “TB evaluation” section (lines 100–106), all patients with clinical suspicion of TB at GHESKIO routinely undergo molecular testing via Xpert MTB/RIF. This highly sensitive diagnostic algorithm allows for a significantly higher rate of bacteriological confirmation compared to standard settings that rely primarily on smear microscopy or clinical diagnosis.

**4. The average frequency of contacts with a healthcare provider in the two models is not listed in the results. Do you have data on this and can it be included?**

**Response:** Thank you for raising this important point. Because this study relied on routine programmatic data rather than a prospective clinical trial design, we unfortunately do not have empirical data on the *actual* average frequency of contacts (such as unscheduled clinic visits or individual call logs) for either the SOC or the DSD cohort. However, we can clarify the *planned* frequency of contacts according to the protocols for each model, which differed significantly by design:

- SOC Model: Patients were scheduled for approximately 7 to 9 in-person clinical visits over the 6-month treatment course (every 2-4 weeks initially, then monthly).
- DSD Model: Patients were scheduled for only 3 in-person visits (initiation, month 2, month 6). This was supplemented by planned remote monitoring (phone/text) biweekly during the first two months and monthly thereafter (approximately 12 remote contacts).

We have added a description of these planned contact frequencies to the Methods section (lines 123-125 and lines 144-147) to better contextualize the interventions.

**5. Line 206 - HIV status did not vary between the two stratum! Did it not? The DSD vs SOC cohorts had 7% and 32% HIV positive! Please correct the statement.**

**Response:** Thank you for highlighting this phrasing, which we agree was easily misinterpreted. We did not mean to imply that HIV prevalence was similar between the DSD and SOC cohorts, as our baseline table clearly demonstrates a significant difference (7.2% vs 31.8%). Rather, we were referring to the statistical mechanics of the stratified analysis. When we built separate regression models specifically for the HIV-positive subset and the HIV-negative subset (the two 'strata'), every patient within a given model had the exact same HIV status. Because HIV status is constant within each specific stratum, it mathematically cannot be included as an adjusting covariate in those specific models. We have revised the text in the paragraph on the sensitivity analyses (line 205) to ensure this methodological step is clear and prevents any confusion regarding the baseline differences between the cohorts.

**6. Reviewer Comment #6: With the significant difference in the proportion of those with successful treatment outcomes, primarily driven also by the LTFU group, would you recommend enhancement in the follow-up strategies of SOC? Please explain the reason why LTFU was excluded from the analyses, as I am unable to understand why this was done.**

**Response:** We agree completely with the reviewer’s insight. The data strongly suggest that the high LTFU rate in the SOC group is the primary driver of the lower treatment success rate. Consequently, enhancing follow-up strategies for the SOC cohort—perhaps by integrating elements of the DSD model, such as routine remote check-ins or more flexible dispensing—is a key programmatic recommendation. We have added text to the Discussion to emphasize this point (lines 354-361).

Regarding the sensitivity analysis on the effect of LTFU, our rationale for excluding LTFU patients in that specific sub-analysis was to determine whether the observed differences in treatment success were driven by the intervention itself or simply by patient retention. Because excluding the LTFU patients eliminated the statistical difference in success rates between the two cohorts, it confirmed that the DSD model's primary mechanism of success is keeping patients in care, rather than a difference in treatment completion outcomes among those who remain in care.

To ensure these points are easily understood, we have revised and reorganized the entire 'Sensitivity Analyses' paragraph in the Methods for clarity (lines 205-217). This includes explicitly detailing the logical rationale for each of our three sensitivity analyses (excluding LTFU, restricting to pulmonary TB, and stratifying by HIV status) to ensure our analytical approach is clear to readers.

**7. Line 311: National rates for successful treatment outcomes are at 85%, whereas in this intervention the rates among those allocated to the SOC group are much lower at 78%. Were the national rates achieved during the COVID-19 pandemic? What could be the reasons for this difference if true?**

**Response:** The reviewer makes an astute observation. The national treatment success rate for Haiti (85%) was indeed reported for 2022, encompassing the ongoing pandemic period. The 78% success rate in our SOC cohort is lower, which we believe is primarily driven by the acute local context and our specific patient population:

1. Acute urban crisis: Our study was conducted in Port-au-Prince, an urban setting that was severely affected by socio-political unrest, extreme gang violence, and a localized humanitarian crisis during the study period. These physical barriers to accessing care disproportionately affected our urban cohort compared to patients in more stable, rural parts of the country.
2. Referral center and patient population: GHESKIO serves as a major national referral center, attracting patients from across the entire country. Consequently, the clinic frequently cares for the sickest individuals and those with complex clinical presentations, particularly complicated TB/HIV co-infections. This highly vulnerable patient population naturally carries a higher baseline risk of poor outcomes and loss to follow-up compared to the general, less complicated TB population reflected in national averages.
3. Selection bias: as previously described, clinician discretion was used to determine who should be offered treatment under the DSD model of care, and the data suggests that “healthier” and more stable patients were offered DSD. Hence individuals in the SOC cohort likely did not represent an average cohort of TB patients in Haiti; many of the “healthier” patients were likely in the DSD group.
4. Data aggregation: National statistics are aggregated over a wider geographic area, which often smooths out the acute, localized drops in treatment success caused by the concentrated crisis in the capital.

We believe these factors combined explain why the SOC baseline success rate at GHESKIO during this period was lower than the overall national average.

**8. Line 316: LTFU rates in the SOC group are 15.5%, but Table 3 shows 16.8%. Which is correct?**

**Response:** Thank you for pointing out this inconsistency. The correct LTFU rate for the SOC cohort is **16.8%**, as shown in Table 3 (67 of 400 patients). The figure of 15.5% on line 314 is a typographical error; it has been corrected in the revised manuscript (Line 320).

**9. Line 328: Do you think that the DSD approach used in this intervention targeted the most-in-need? Would the most-in-need not be those with co-morbidities, lower body weight, etc.,? I think this should be a recommendation, rather than a conclusion as this intervention does not appear to have targeted the most-in-need.**

**Response:** The reviewer makes an excellent and highly insightful point. We completely agree: the DSD model as implemented in this cohort targeted clinically stable patients who were inherently “less in need” of intensive clinical management (e.g., fewer HIV co-infections, higher body weight). The “most-in-need” patients were those who remained in the SOC cohort. Our original phrasing did not capture this distinction clearly. The true strength of the differentiated care model is that by shifting stable patients into less intensive models, the health system is able to redirect its limited, high-intensity resources toward those “most-in-need” patients remaining in standard care. We have revised our conclusion in the Discussion section (lines 357-361) to reflect this critical nuance, clarifying that the broader DSD framework allows systems to focus resources on the most vulnerable, rather than implying that this specific DSD intervention itself targeted them.

## **Editor’s Comment**

**Editor Comment: I agree with the reviewer's comments / perspective. I wonder if lines 314-328 are somewhat duplicative of the limitations sub-section. I suggest that this could be resolved if the discussion is re-organised such that the first paragraph stays as is, followed by a paragraph sub-headed 'strengths and limitations' and then the content of lines 314-328 and current limitations are folded together under this. To then continue with what is currently paragraph 3 and onwards under the sub-heading 'contribution to literature'. Lastly, that should create additional space for more specific recommendations for policy / practice and future research (see reviewer's comments).**

**Response:** We sincerely thank the Editor for this helpful suggestion. As requested, we have fully merged the discussion of LTFU (formerly lines 314-328) into the limitations section, which successfully eliminated the previous duplication.

Regarding the structural flow, we found that using formal subheadings and placing the limitations immediately after the opening paragraph interrupted the natural transition into the broader literature. To maintain a standard, cohesive scientific narrative, we presented the revised Discussion without subheadings and placed the newly merged limitations paragraph toward the end of the section.

Finally, we utilized the space saved to add a dedicated concluding paragraph on policy, practice, and future research. Please note that to comply with our agency’s clearance guidelines, we framed these as 'considerations' rather than explicit 'recommendations' or 'should' statements. We believe these revisions successfully address the structural concerns while preserving the flow of the manuscript.
